# High-resolution monochromator for the 8.41 keV ^169^Tm nuclear resonance

**DOI:** 10.1107/S1600577526009112

**Published:** 2026-10-27

**Authors:** Sven Velten, Deepak Prajapat, Frank-Uwe Dill, Ilya Sergeev

**Affiliations:** a Deutsches Elektronen-Synchrotron DESY Notkestr. 85 22607Hamburg Germany; Australian Synchrotron, Australia

**Keywords:** rare-earth materials, ^169^Tm Mössbauer spectroscopy, high-resolution monochromators, nuclear resonant scattering, PETRA III, hyperfine parameters

## Abstract

Rare-earth elements are key components in a wide range of functional materials, including solid-state lasers, superconductors and high-performance permanent magnets, owing to their localized 4*f* electrons that give rise to distinctive electronic and magnetic properties. Nuclear resonant scattering of synchrotron radiation using Mössbauer isotopes provides a powerful isotope-specific probe of these properties, enabling access to vibrational dynamics as well as electronic and magnetic hyperfine interactions. Its application, however, requires dedicated high-resolution monochromators tailored to the ultra-narrow nuclear resonances of individual Mössbauer isotopes. The 8.41 keV nuclear resonance of ^169^Tm was among the first resonances excited at synchrotron sources, but has not been revisited since the early pioneering experiments. Here, a narrow-band high-resolution monochromator optimized for the ^169^Tm nuclear resonance at the High-Resolution Dynamics Beamline P01 of PETRA III (DESY, Hamburg) is presented. The monochromator provides a tunable energy resolution between 1.4 and 0.33 meV. Its performance is demonstrated by nuclear forward scattering and nuclear inelastic scattering measurements on Tm_2_O_3_ and metallic Tm, allowing the determination of hyperfine parameters and the phonon density of states, and derived thermodynamic quantities. The instrument establishes ^169^Tm nuclear resonant scattering as a probe of vibrational, electronic and magnetic properties in Tm-based materials.

## Abstract 2

A high-resolution monochromator for the 8.41 keV ^169^Tm nuclear resonance is presented enabling nuclear resonant scattering on Tm compounds at PETRA III beamline P01. Hyperfine parameters and phonon densities of states of metallic Tm and Tm_2_O_3_ are studied.

## 1. Introduction

Nuclear resonant scattering (NRS) using synchrotron radiation provides a powerful probe of hyperfine interactions and lattice dynamics through resonant nuclear absorption of X-rays (Gerdau & de Waard, 1999 ▸, 2000 ▸). However, the extremely narrow natural linewidths of nuclear resonances, typically of the order of nanoelectronvolts, pose severe challenges for synchrotron-based experiments. Typical Si high-heat-load monochromators at synchrotron beamlines provide an energy bandwidth of a few electronvolts down to 100 meV, resulting in an X-ray beam dominated by non-resonant photons. These photons completely obscure the much weaker resonant signal in the photon detector. Consequently, additional monochromatization is required, motivating the development of specialized high-resolution monochromators (HRMs) tailored to specific nuclear resonances.

Historically, the 8.41 keV nuclear resonance of ^169^Tm was the second nuclear resonance explored at synchrotrons (Sturhahn *et al.*, 1991 ▸). This was motivated by ^169^Tm being the only stable thulium isotope and thus having a 100% natural isotopic abundance, eliminating the need for costly isotopic enrichment (Sturhahn *et al.*, 1991 ▸; Leupold *et al.*, 1999 ▸). Furthermore, conventional ^169^Tm Mössbauer spectroscopy suffers from large background noise, the short half-life of the source, and a typically broadened source linewidth that limits spectral resolution (Sturhahn *et al.*, 1991 ▸). The early experiments employed a Si(444) channel-cut monochromator with an energy resolution of ∼90 meV to record the nuclear resonance signal (Sturhahn *et al.*, 1991 ▸). However, the relatively large natural linewidth of 114 neV, corresponding to a short lifetime of 5.9 ns, together with the limited time resolution of the detectors and the lower brilliance of early synchrotron sources, significantly constrained the practical use of the ^169^Tm resonance. Subsequent advances in X-ray optics, detector technology and synchrotron facilities ultimately favored other isotopes such as ^57^Fe for NRS experiments (Leupold *et al.*, 1999 ▸).

Since those early experiments, NRS has been extended to a wide range of Mössbauer isotopes, including a few rare-earth elements such as ^149^Sm (Kitao *et al.*, 2000 ▸), ^151^Eu (Leupold *et al.*, 1996 ▸), ^161^Dy (Shvyd’ko *et al.*, 2001 ▸; Chumakov *et al.*, 2001 ▸) or ^45^Sc (Shvyd’ko *et al.*, 2023 ▸). The great development of X-ray optics and detectors now enables the excitation of very short-lived nuclear resonances, such as ^129^Xe (1.5 ns) (Klobes *et al.*, 2013 ▸), as well as extremely long-lived resonances with their ultra-narrow transition linewidths, *e.g.*^45^Sc with lifetimes around 0.5 s. However, the ^169^Tm resonance has not been revisited in NRS since these early studies, despite the increasing use of thulium in several fields. In particular, Tm-doped solid-state lasers have gained considerable relevance, *e.g.* for medical applications (Xu *et al.*, 2025 ▸) or for gas detection (Loiko *et al.*, 2021 ▸). In these systems, the Tm laser transition is strongly influenced by phonon processes (Loiko *et al.*, 2021 ▸), making experimental access to the phonon behavior at the Tm site highly desirable. In addition, the localized 4*f* electrons of Tm carry a comparatively large magnetic moment, which is exploited in Tm-based magnetic materials, including superconductors (Huang *et al.*, 2024 ▸), permanent rare-earth magnets (Tereshina *et al.*, 2018 ▸) and single-molecule magnets (Harriman *et al.*, 2017 ▸).

Here, we present a narrow-band HRM at beamline P01 at PETRA III (DESY, Hamburg) for the ^169^Tm resonance. The HRM achieves an energy resolution of 1.4 meV. Because of its dispersive nature, energy resolutions down to 0.3 meV are possible as well. This enables studies of hyperfine interactions, for example of magnetic structures in Tm compounds, as well as investigations of lattice dynamics at the Tm site, including measurements of the phonon density of states (PDOS). As a demonstration, we performed NRS experiments on Tm_2_O_3_ and Tm metal.

## 2. Setup

**Figure 1:**
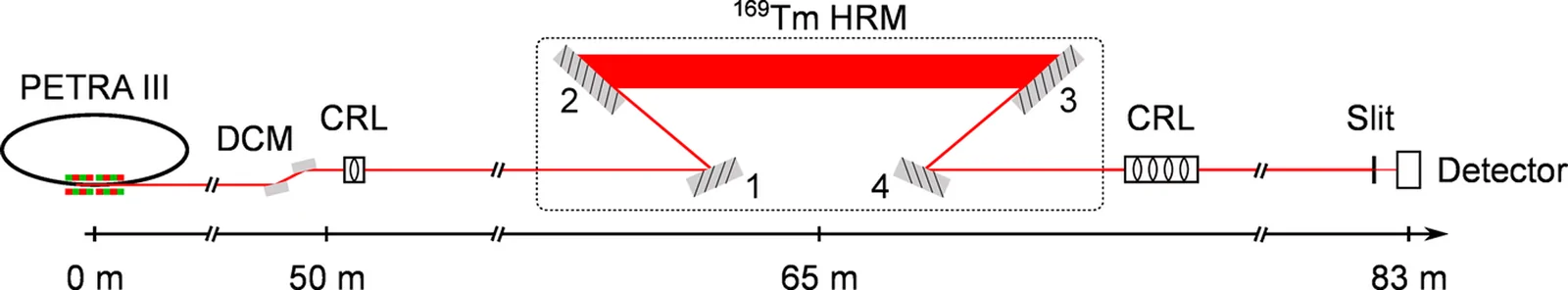
Schematic drawing of the ^169^Tm HRM setup at PETRA III. Positions of double-crystal monochromator (DCM) and compound refractive lenses (CRLs) are given relative to the central undulator position. The HRM schematic drawing depicts the orientation of the atomic (111) planes in the Si crystals and the effect of the asymmetrical cut crystals on the beam width.

The schematic setup is depicted in Fig. 1 ▸. Two 5 m undulators and a high-heat-load double-crystal monochromator (DCM) using two Si(111) reflections result in an initial monochromatization of the incident X-rays to a bandwidth of 1 eV. The exiting angular divergence of ∼10 µrad has to be reduced to render an effective monochromatization afterwards. Thus, directly after the DCM (∼50 m from the undulator source), a parabolic Be lens with a curvature radius of 0.5 mm is placed in the beam to reduce the angular divergence to ∼3 µrad.

The center energy of the undulators and DCM was tuned to the nuclear resonance by searching for the nuclear resonance fluorescence signal from a thick Tm_2_O_3_ powder absorber. An initial energy calibration was conducted using the *K* edge of nickel at 8.33149 (2) keV (Kraft *et al.*, 1996 ▸). With that, the nuclear resonance was found at 8.410 (1) keV, perfectly matching the reported resonance energy of 8.41017 (11) keV (Baglin, 2008 ▸).

At 15 m distance from the DCM, the new HRM is placed. It uses four Si(444) reflections in a (+ − − +) reflection geometry, similar to the setup of Yabashi *et al.* (2001 ▸). The four crystals have different asymmetry parameters, with 1/*b*_1_ = *b*_4_ = −2.2055 (corresponding to an asymmetry angle of ±46.10°) and 1/*b*_2_ = *b*_3_ = −11.3378 (corresponding to ±66.64°), such that *b*_1_*b*_2_ = −1/25. The beam width therefore increases by a factor of 25 after the first two reflections. The first crystal has an acceptance angular divergence of ∼20 µrad rendering a full beam acceptance. The crystals are 10 mm thick and 30 mm wide, with lengths along the beam direction of either 40 mm for crystals 1 and 4 or 60 mm for crystals 2 and 3, accommodating the increased beam footprint on the inner crystals. To optimize their performance, the crystal surfaces underwent chemical-mechanical polishing for one week. The crystals are pairwise mounted on two Kohzu high-precision goniometers (KTG-15D). The first and last crystals are also mounted on Attocube double tilt stages (ECG5050). All angular motors have a precision of below 20 nrad, allowing for a high-precision angular alignment of each crystal. Both crystal pairs are placed in a He-filled box to reduce air absorption to less than one percent.

## 3. Results

**Figure 2:**
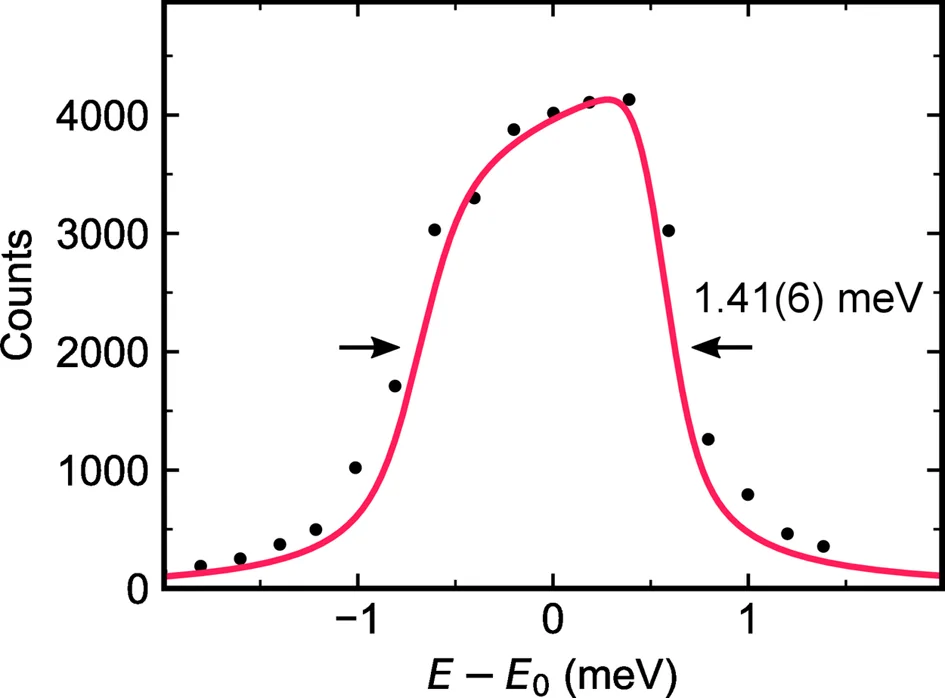
Measured instrumental function of the ^169^Tm HRM in comparison with simulation (red line).

To measure the instrumental function, the transmission through a thin layer (∼5 µm) of Tm_2_O_3_ powder was measured. The delayed resonance signal from the nuclear decay was detected by two avalanche photodiodes (APDs), each consisting of a 100 µm Si layer, in a time window starting from 10 ns after X-ray excitation. The latter ensures that no non-resonant signal is detected. The scan of the HRM energy across the nuclear resonance is achieved by a combined angular motion of all four crystals and is shown in Fig. 2 ▸. A full width at half-maximum (FWHM) of 1.41 (6) meV was achieved. The asymmetric shape originates from absorption effects within the Darwin plateau and is reproduced by simulations using the dynamical theory of diffraction (Shvyd’ko, 2004 ▸, 2015 ▸), as shown in Fig. 2 ▸. The slight deviation from the simulated FWHM of 1.3 meV can be attributed to slight bendings and/or miscuts of the crystal surfaces, especially considering the relatively large beam footprint of 29 mm (FWHM) on the second and third crystals (assuming a vertical beam height FWHM of 0.8 mm after the DCM). The total photon flux measured through the HRM is 1.0 (2) × 10^10^ photons s^−1^ meV^−1^.

The instrumental function can be narrowed if needed by exploiting the dispersion arising from the specific HRM layout. The combined dispersion after four consecutive Bragg reflections is given by (Shvyd’ko *et al.*, 2013 ▸; Shvyd’ko, 2015 ▸; Chumakov *et al.*, 2019 ▸) 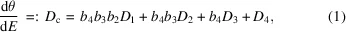
with
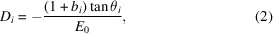
with *b*_*i*_ being the asymmetry parameter of each crystal, θ_*i*_ being the Bragg angle (= 70.1047° in the kinematical approximation for all crystals) and *E*_0_ = 8.41 keV being the resonance energy. Following equation (1) the combined dispersion is *D*_c_ = −15.77 µrad meV^−1^. To utilize the dispersion as a means to narrow down the instrumental function, a focal lens is required to focus the different wavelengths to different vertical positions. Thereby, the smallest possible energy resolution achievable is given by (Shvyd’ko, 2015 ▸; Chumakov *et al.*, 2019 ▸) 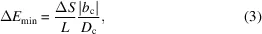
with the effective source size Δ*S*, the distance of source-to-HRM *L* and the total asymmetry parameter |*b*_c_| = |*b*_4_*b*_3_*b*_2_*b*_1_| (= 1 in the given case).

We evaluated the dispersive nature of the HRM by focusing the beam with a Be compound refractive lens (CRL) placed directly after the HRM to the detector located at 18 m distance from the HRM. The focal size of the monochromatic beam (NRS signal) was 65 µm (FWHM). Assuming a linear lens magnification, the effective vertical source size can be estimated by Δ*S* = *h*_*i*_*d*_*i*_/*L*. With the image distance *d*_*i*_ = 18 m, image size *h*_*i*_ = 65 µm and *L* = 50 m (assuming a collimated beam downstream of the first CRL after the DCM), an effective vertical source size of ∼175 µm can be estimated.

**Figure 3:**
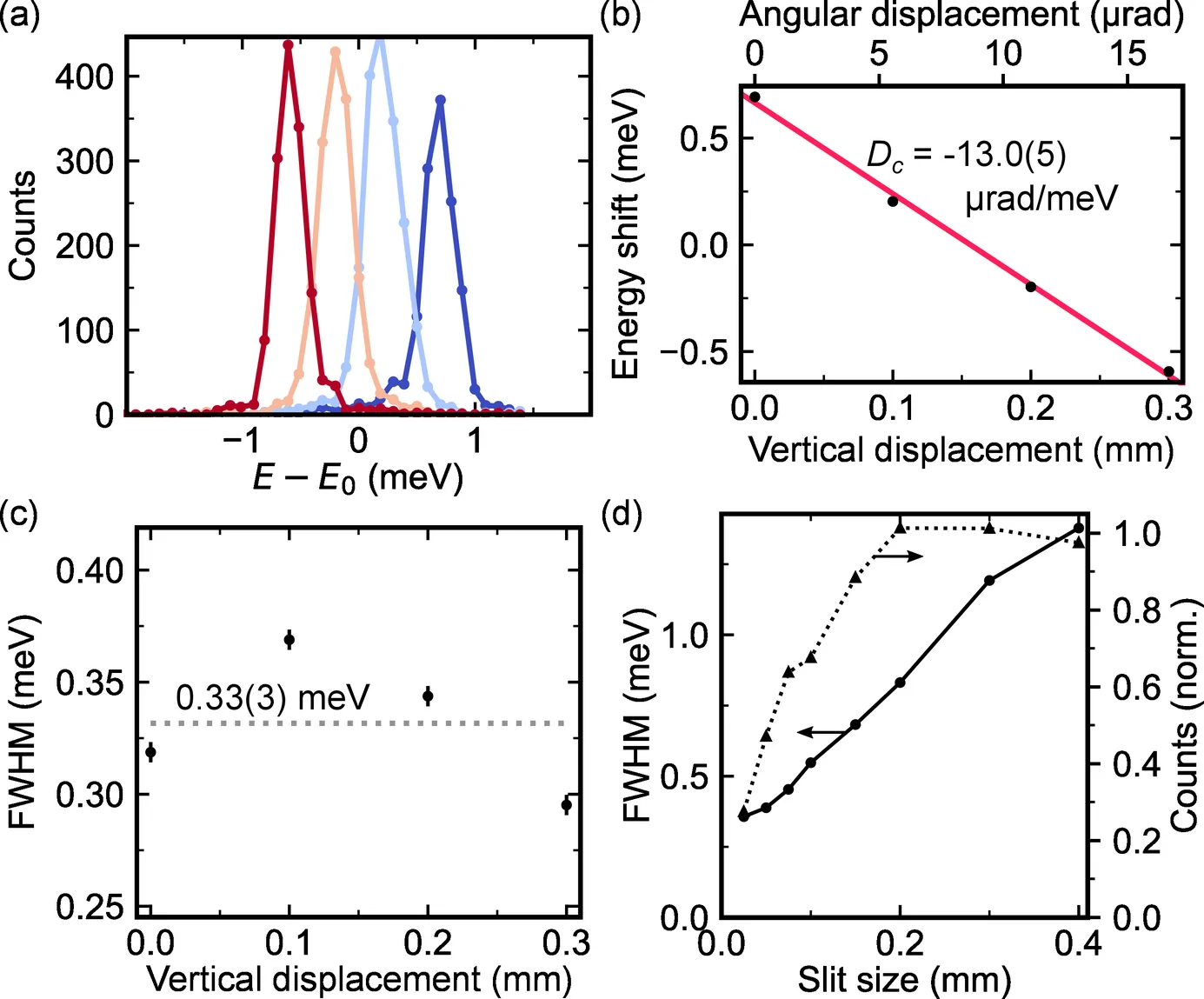
(*a*) In combination with lenses, the focal spots of different wavelengths within the instrumental function are angular displaced due to the dispersive nature of the HRM (here measured at four different spots by the vertical displacement at 18 m distance from the HRM using a 25 µm slit in front of the detector). (*b*) A linear regression (red line) of the energy shift as a function of the angular displacement reveals the combined dispersion *D*_c_. (*c*) Derived FWHM at each of the four detector positions. The average FWHM is denoted by the dotted line. (*d*) FWHM (left axis, solid circles) and nuclear resonant counts (right axis, dashed triangles) as a function of the slit size in front of the detector. The resonant counts are normalized to the average maximum value with open slits.

By using an adjustable slit in front of the detector, we select a specific energy band from the dispersive spectrum and the instrumental function can be reduced to Δ*E*_min_. As shown in Fig. 3 ▸(*a*) using a 25 µm slit, the central energy of the instrumental function depends on the vertical displacement of the detector, while its width at each position is substantially narrowed. The evaluation of the energy shift as a function of the vertical displacement is depicted in Fig. 3 ▸(*b*) and reveals a dispersion of −13.0 (5) µrad meV^−1^. The FWHM at each vertical displacement is roughly constant with a mean value of 0.33 (3) meV [Fig. 3 ▸(*c*)].

Furthermore, the width of the instrumental function can be tuned by the slit size, as shown in Fig. 3 ▸(*d*); however, it is ultimately limited by equation (3). This limit appears to be reached at a slit size of 25 µm. Using the estimated effective source size of Δ*S* = 175 µm, a minimal energy resolution of 0.27 meV could be achieved, which is close to the measured 0.33 (3) meV. At the same time, the NRS signal depicted in Fig. 3 ▸(*d*) remains constant until the slit starts to clip the focused monochromatic beam with its FWHM of 65 µm.

The achieved sub-meV resolution enables high-precision studies of phonon properties using nuclear inelastic scattering. Moreover, a much lower resolution is theoretically possible, but imperfect optics appear to limit Δ*E*_min_. The effective vertical source size of 175 µm is drastically increased from the nominal value of 15 µm. This broadening strongly indicates perturbations of the beam properties by the two monochromators and the lenses. Especially a possible bending of the inner two HRM crystals, as already discussed for the unfocused case, can easily cause this deviation. This is also in accordance with the 13% lower measured dispersion relative to the theoretical dispersion, as a slight crystal bending can easily change the dispersion as well.

As an application, we studied Tm_2_O_3_ and Tm metal using nuclear forward scattering and nuclear inelastic scattering with unfocused X-rays to extract hyperfine parameters and lattice dynamics.

### 3.1. Nuclear resonant scattering study of Tm_2_O_3_

Tm_2_O_3_ has the rare-earth-typical sesquioxide (*R*_2_O_3_) structure. At ambient conditions, it finds itself in the cubic phase where Tm atoms occupy two different crystal sites exhibiting different point symmetries, C_2_ and C_3*i*_, with the relative weight of 3:1. Early Mössbauer studies found that both sites exhibit a quadrupole splitting and a small relative isomer shift (Kalvius, 1962 ▸; Stewart *et al.*, 1988 ▸).

**Figure 4:**
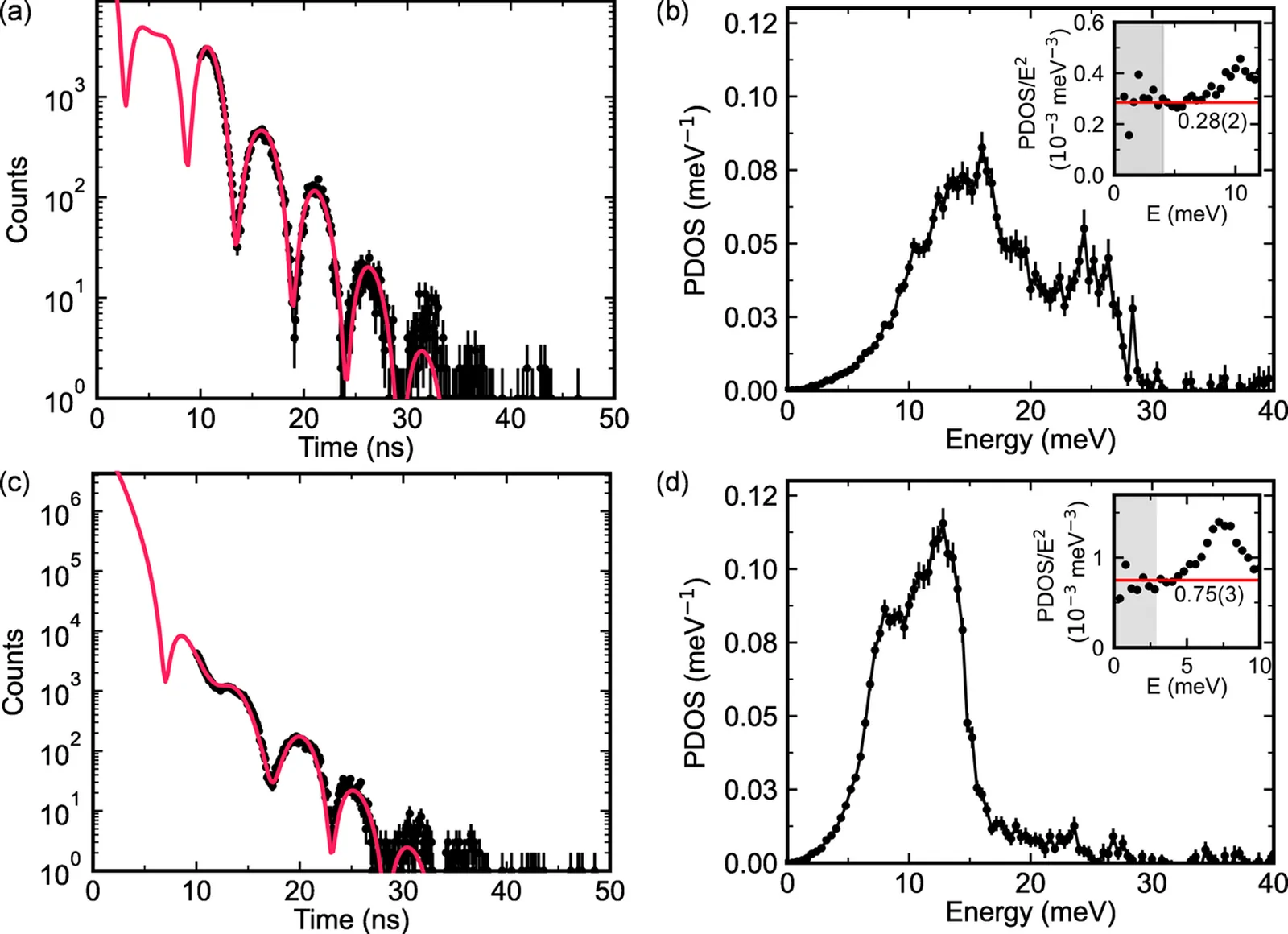
Nuclear forward and nuclear inelastic scattering results. (*a*) Time-dependent nuclear forward decay following X-ray excitation and (*b*) PDOS from a thinly spread Tm_2_O_3_ powder (∼4 µm). (*c*) Nuclear forward decay and (*d*) PDOS from a 45° inclined 10 µm Tm metal foil. The decay patterns were recorded 10 ns after X-ray excitation. The fit (red lines) allows one to extract the hyperfine parameters. The insets in (*b*) and (*d*) show the PDOS divided by the square of the energy, allowing the Debye level to be derived (red line). The gray shaded region is neglected in the calculation due to potential artefacts from the elastic line removal. The Debye-like behavior was assumed to be up to 9 meV for Tm_2_O_3_ and 5 meV for Tm metal.

The time-dependent nuclear decay in the forward direction following X-ray excitation is shown in Fig. 4 ▸(*a*). It exhibits mainly a single-frequency oscillation. The spectrum was analyzed using *Nexus* (Bocklage, 2024 ▸) and *CONUSS* (Sturhahn, 2020 ▸) and the results are listed in Table 1 ▸. The oscillation corresponds to a quadrupole splitting of 28.2 (5) mm s^−1^ [= 7.0 (1) Γ_0_, with Γ_0_ = 114 neV]. A slightly better fit of the decay pattern is found when introducing a second site with a quadrupole splitting corresponding to the C_3*i*_ Tm site. The relative weight was fixed at 3:1. For the C_3*i*_ site, a quadrupole splitting of 57.2 (5) mm s^−1^ [= 14.1 (1) Γ_0_] is derived with zero isomer shift (within the error bar). The magnitude of both splittings corresponds very well to the results of the early Mössbauer studies (Kalvius, 1962 ▸; Stewart *et al.*, 1988 ▸). The C_3*i*_ site lines appear to be substantially broader indicating that Tm atoms at this site might experience larger variations of field gradients due to distortions.

From the nuclear inelastic data, the (partial) PDOS of Tm can be derived. Using the software package *nisdos* (developed and provided by Sergeev), the partial PDOS of Tm inside Tm_2_O_3_ is obtained, depicted in Fig. 4 ▸(*b*). A Debye-like behavior can be observed until 9 meV. Peaks around 16 and 24 meV indicate flat phonon dispersion bands at the respective energies, and a cut-off energy of 29 meV is derived by the falling edge of the PDOS. From the PDOS, the Lamb–Mössbauer factor and the vibrational contribution to a couple of thermodynamic quantities can be derived (Chumakov & Sturhahn, 1999 ▸). They are listed in Table 2 ▸. The Lamb–Mössbauer factor *f*_LM_ is 0.926 (3) at room temperature. Such a high *f*_LM_ is typical for Mössbauer isotopes with relatively low resonance energies because of the correspondingly lower recoil energy (for ^169^Tm: 0.23 meV; in comparison, for ^57^Fe: 2.0 meV). Furthermore, the lattice bonding in oxides is typically strong, leading to an increased *f*_LM_.

From the initial quadratic slope of the PDOS, the Debye speed of sound *v*_D_ can be derived by (Chumakov *et al.*, 2009 ▸) 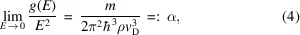
where *g*(*E*) denotes the (partial) PDOS, *m* is the mass of the resonant atom (*m*_Tm_ = 168.934 u) and ρ is the mass density of the material. With α = 0.28 (2) × 10^−3^ meV^−3^ [see inset in Fig. 4 ▸(*b*)] and ρ = 8.53 g cm^−3^, *v*_D_ = 2740 (50) m s^−1^ is obtained. This can be compared with the Debye speed derived from elastic constants measured for Tm_2_O_3_. From the sheer modulus *G* and the Young modulus *E*, and assuming that the material is elastically isotropic, the transversal and longitudinal speed of velocity, *v*_t_ and *v*_l_, are given by (Rosen, 1968 ▸)
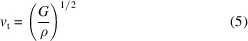
and
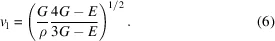
The Debye speed *v*_D_ is then the averaged speed, given by (Rosen, 1968 ▸) 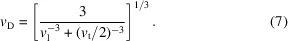
Using *G* = 62.91 GPa and *E* = 162.54 GPa as reported for Tm_2_O_3_ at room temperature (Manning & Hunter Jr, 1970 ▸), a Debye speed of 3030 m s^−1^ is derived. The small discrepancy to the value derived here can be attributed to the underlying assumptions of equations (5)–(7), namely a harmonic and isotropic material, which might not be strictly valid for Tm_2_O_3_.

### 3.2. Nuclear resonant scattering study of Tm metal

The time-dependent nuclear forward decay from elemental Tm metal is shown in Fig. 4 ▸(*c*). At room temperature, Tm metal is paramagnetic and has a hexagonal-closed-packed (hcp) crystal structure. Thus, the hyperfine structure is not expected to exhibit any magnetic hyperfine splitting, but a quadrupole splitting cannot be excluded. The recorded nuclear decay was therefore fit using one hyperfine site with a potential quadrupole splitting. Additionally, an oxide layer was assumed. The natural oxide layer was modeled by a Tm_2_O_3_ layer with hyperfine parameters as previously determined. The result is depicted in Fig. 4 ▸(*c*) and the found hyperfine parameters are listed in Table 3 ▸. We found ∼7% of Tm_2_O_3_ in the Tm metal foil. For metallic Tm, the fit yields a quadrupole splitting of only 3 (9) mm s^−1^ [∼1 (2) Γ_0_] and an isomer shift relative to Tm_2_O_3_ of only 1 (1) mm s^−1^ [= 0.2 (2) Γ_0_]. The sign of the isomer shift cannot be derived from this measurement. Within the uncertainties, both values are compatible with zero. These results are in agreement with early Mössbauer studies showing a broad single line for Tm metal (Uhrich *et al.*, 1968 ▸).

The PDOS derived from the nuclear inelastic scattering data is shown in Fig. 4 ▸(*d*). Compared with Tm_2_O_3_, the spectrum is shifted towards lower energies. A Debye-like regime is observed up to 5 meV, followed by pronounced features at 8 and 13 meV, and the phonon spectrum rapidly terminates at 15 meV. However, there are still phonon modes present at higher energies up to ∼30 meV. The comparison with the PDOS of Tm_2_O_3_ strongly suggests that these high-energy modes originate from the thin oxide layer, consistent with results from the nuclear forward decay pattern. The thermodynamic quantities derived from the PDOS are summarized in Table 2 ▸. The resulting *f*_LM_ of 0.858 (6) is still relatively high, as expected (see discussion above). From the quadratic Debye behavior below 5 meV, a Debye speed of sound of 1930 (30) m s^−1^ is obtained from equation (4) with α = 0.75 (3) × 10^−3^ meV^−3^ [see inset in Fig. 4 ▸(*d*)] and ρ = 9.3 g cm^−3^. For comparison, using elastic constants *G* = 30.5 GPa and *E* = 74.0 GPa, as reported for Tm metal at room temperature (Rosen, 1971 ▸), a Debye speed of 2002 m s^−1^ is derived from equations (5)–(7). Similar to the Tm_2_O_3_ case, the Debye speed reported here is close to, but slightly smaller than, the value derived from elastic constants, possibly due to the same reasons discussed above.

## 4. Conclusions

In summary, we have demonstrated a novel HRM for the Mössbauer isotope ^169^Tm. With an energy resolution of 1.4 meV in the unfocused configuration, down to 0.33 meV using focusing optics, the ^169^Tm nuclear resonance at 8.41 keV can be efficiently excited. This enables high-resolution measurements of hyperfine interactions and lattice dynamics, as demonstrated for Tm_2_O_3_ and Tm metal.

The minimal energy resolution could be further reduced by improving the crystal quality of the HRM, particularly of the second and third crystals, which are most susceptible to bending effects. Using the nominal source size of 15 µm, the minimum achievable resolution is ∼0.02 meV. Furthermore, the start time for collecting the nuclear signal could be improved. At 8.4 keV, a 100 µm Si APD absorbs more than 75% of the incident beam, leading to rapid detector overload even at moderate beam intensities and resulting in measurement artefacts up to 8 ns after X-ray excitation. While thinner APDs are not commercially available at the moment, the load effect can be mitigated in special sample or setup geometries, such as nuclear Bragg diffraction, grazing incidence scattering of thin films or by using X-ray polarization filtering to detect only X-rays with a rotated polarization caused by the nuclear interaction.

The new HRM provides a powerful tool to disentangle vibrational, magnetic and electronic properties, *e.g.* in magnetic compounds or Tm-based solid-state lasers.

## Figures and Tables

**Table 1:** Hyperfine parameters (δ: isomer shift; Δ*E*_Q_: quadrupole splitting) found for Tm_2_O_3_ The powder was fit with a Gaussian thickness distribution with the center at 4.7 (1) µm and an FWHM of 5.1 (3) µm. Parameters that were kept fixed during the fit routine are indicated as such. The isomer shift is relative to the C_2_ site. For conversion: 1 mm s^−1^ = 0.247 Γ_0_ = 28.1 neV.

Site	|δ| (mm s^−1^)	Δ*E*_Q_ (mm s^−1^)	FWHM (mm s^−1^)
C_2_	0 (fixed)	28.2 (5)	4.0 (5)
C_3*i*_	0 (1)	57.2 (5)	14.0 (5)

**Table 2:** Lamb–Mössbauer factor *f*_LM_, vibrational contribution to the internal energy *U*, mean force constant *K*, lattice specific heat at constant volume *c*_V_, vibrational entropy *S*_vib_ and the Debye speed of sound *v*_D_, derived from the PDOS for Tm_2_O_3_ and Tm metal measured at 296 K

Material	*f*_LM_	*U* (meV)	*K* (Nm^−1^)	*c*_V_ (3*k*_B_)	*S*_vib_ (3*k*_B_)	*v*_D_ (m s^−1^)
Tm_2_O_3_	0.93 (1)	80 (3)	207 (9)	0.97 (3)	1.51 (5)	2740 (50)
Tm metal	0.86 (1)	77 (2)	96 (5)	0.94 (3)	1.82 (6)	1930 (30)

**Table 3:** Hyperfine parameters (δ: isomer shift; Δ*E*_Q_: quadrupole splitting) found for the 10 µm Tm metal foil The foil was inclined by ∼45°. The oxide layer was modeled by a Tm_2_O_3_ layer using the previously found hyperfine parameters (see Table 1 ▸). Parameters that were kept fixed during the fit routine are indicated as such. The isomer shift is relative to Tm_2_O_3_. For conversion: 1 mm s^−1^ = 0.247 Γ_0_ = 28.1 neV.

Compound	Thickness (µm)	Site	|δ| (mm s^−1^)	Δ*E*_Q_ (mm s^−1^)	FWHM (mm s^−1^)
Tm metal	13.7 (6)	–	1 (1)	3 (9)	12 (3)
Tm_2_O_3_	0.9 (1)	C_2_	0 (fixed)	28.2 (fixed)	7 (1)
		C_3*i*_	0 (fixed)	57.2 (fixed)	13 (1)

## Data Availability

The data that support the findings of this study are available from the corresponding author upon request.
